# The effects of a human food additive, titanium dioxide nanoparticles E171, on *Drosophila melanogaster* - a 20 generation dietary exposure experiment

**DOI:** 10.1038/s41598-018-36174-w

**Published:** 2018-12-18

**Authors:** Boris Jovanović, Nikola Jovanović, Vladimir J. Cvetković, Sanja Matić, Snežana Stanić, Elizabeth M. Whitley, Tatjana Lj. Mitrović

**Affiliations:** 1 Department of Natural Resource Ecology and Management, Iowa State University, Ames, IA USA; 2 Department of Biology and Ecology, Faculty of Sciences and Mathematics, University of Niš, Niš, Serbia; 3 Department of Biology and Ecology, Faculty of Science, University of Kragujevac, Kragujevac, Serbia; 4 Pathogenesis, LLC, Gainesville, FL USA

## Abstract

In this study, fruit flies (*Drosophila melanogaster*) were exposed to an estimated daily human E171 consumption concentration for 20 generations. Exposure to E171 resulted in: a change in normal developmental and reproductive dynamics, reduced fecundity after repetitive breeding, increased genotoxicity, the appearance of aberrant phenotypes and morphologic changes to the adult fat body. Marks of adaptive evolution and directional selection were also exhibited. The larval stages were at a higher risk of sustaining damage from E171 as they had a slower elimination rate of TiO_2_ compared to the adults. This is particularly worrisome, since among the human population, children tend to consume higher daily concentrations of E171 than do adults. The genotoxic effect of E171 was statistically higher in each subsequent generation compared to the previous one. Aberrant phenotypes were likely caused by developmental defects induced by E171, and were not mutations, since the phenotypic features were not transferred to any progeny even after 5 generations of consecutive crossbreeding. Therefore, exposure to E171 during the early developmental period carries a higher risk of toxicity. The fact that the daily human consumption concentration of E171 interferes with and influences fruit fly physiological, ontogenetic, genotoxic, and adaptive processes certainly raises safety concerns.

## Introduction

Since 1916, more than 200,000,000 metric tons of titanium dioxide (TiO_2_) have been produced worldwide, of which a significant amount is used as an inactive human food ingredient, E171^1^. Although E171 is not a nanomaterial *per se*, and industrial producers are not required to report it as a nanosized product^2^, E171 contains anywhere between 17–36% of nanoparticles^3–5^. The daily estimated average human consumption of food grade E171 TiO_2_ is 0.2 to 2 mg kg^−1^ body weight (bw)^4^. Chewing a single piece of bubblegum can result in an intake of over 5 mg of E171^6^. The estimated daily maximum consumption of E171 by children is up to 32.4 mg kg^−1^ bw per day^7^. Recent evidence suggests that E171 can translocate across the human gut^8^, is absorbed into the bloodstream of humans^9^, and is likely deleterious to human health^1,10,11^. Although a recent human safety re-evaluation of E171 was performed by the European Food Safety Authority (EFSA), not many new data were considered, and absence of multigenerational studies with reproductive endpoints was noted^7^.

The fruit fly, *Drosophila melanogaster*, is a common model species used in human health research, with many conserved biological, physiological, toxicological, or neurological responses to stimuli, in addition to conserved disease pathways, homologous genetics, short generation time, and low comparative cost^12,13^. The use of *D*. *melanogaster* in nanotoxicology research has gained rapid momentum in the last few years^14–16^. Previously, we showed that dietary exposure of *D*. *melanogaster* to E171 leads to a significant increase in pupation time, down-regulation of the genes involved in oxidative stress, and occasional aberrant phenotypes^17^. However, this was a preliminary study in which only the larvae of a single generation were fed with various concentrations of E171 in order to detect the effects after subchronic exposure. In a different study, an F_2_ generation of water fleas whose parents were previously exposed to TiO_2_ exhibited a significantly higher sensitivity to TiO_2_ compared with the offspring of unexposed adults^18^. As E171 has been used as an inactive human food ingredient since 1966^1^, enough time has passed by now for several human filial generations to have been exposed. Therefore, the aim of the present research is to assess the dietary exposure of 20 subsequent generations of *D*. *melanogaster* (through its entire life cycle of larvae and adults) to an estimated daily human consumption concentration of E171 TiO_2_. During the exposure, the effects of E171 on fecundity, egg to adult viability, developmental time, genotoxicity, morphology, frequency of phenotype aberrations, and breeding dynamics were monitored.

## Results

**Figure 1:**
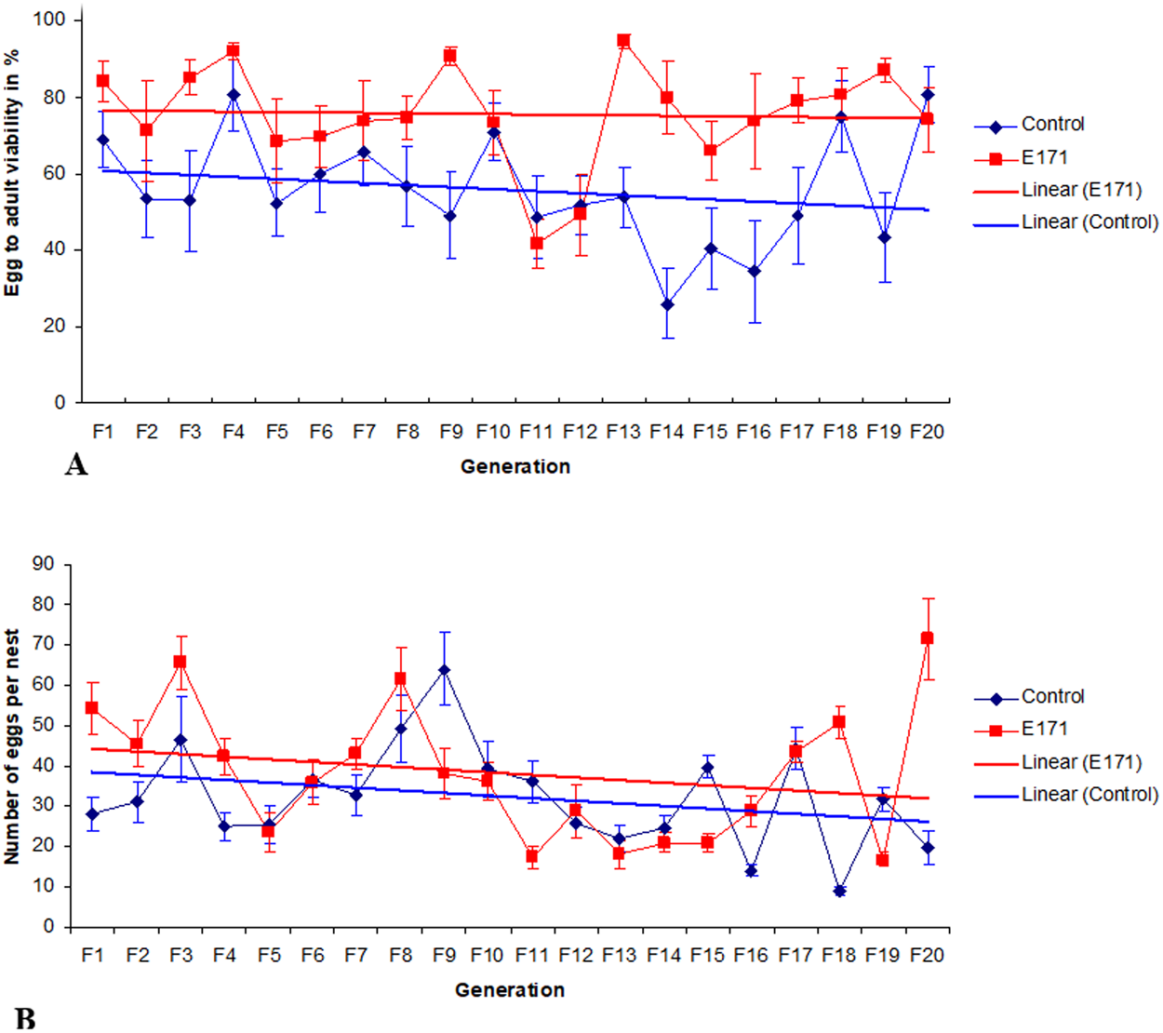
(**A**) Egg to adult viability across 20 generations of *D*. *melanogaster* fed with E171. (**B**) *D*. *melanogaster* fecundity across 20 generations fed with E171. For each generation the egg to adult viability/fecundity was calculated for the first batch of deposited eggs by virgin females. The parents were approximately 6-7 days post eclosion old. Whiskers represent the standard error of the mean.

The egg-to-adult viability of offspring of virgin females was strongly affected by the treatment, as evidenced by a much higher percentage in the E171-exposed cohort. The egg-to-adult viability over the 20 generations in the control was 55.16 ± 4.68% (mean ± 95% Confidence Interval (CI)), while in the E171 treated group, it was 75.33 ± 3.74% (mean ± 95% CI) (Mann-Whitney U-test: p ≪ 0.001; N = 193 per group). This effect was also persistent within the generations (RANOVA: F = 51.8 and F = 2.12; p < 0.001 and p < 0.01 for the treatment and generation number, respectively) as outlined in Fig. 1A. In nearly all generations, the egg-to-adult viability of offspring of virgin females was significantly higher in the E171 treatment compared to the control.

**Figure 2:**
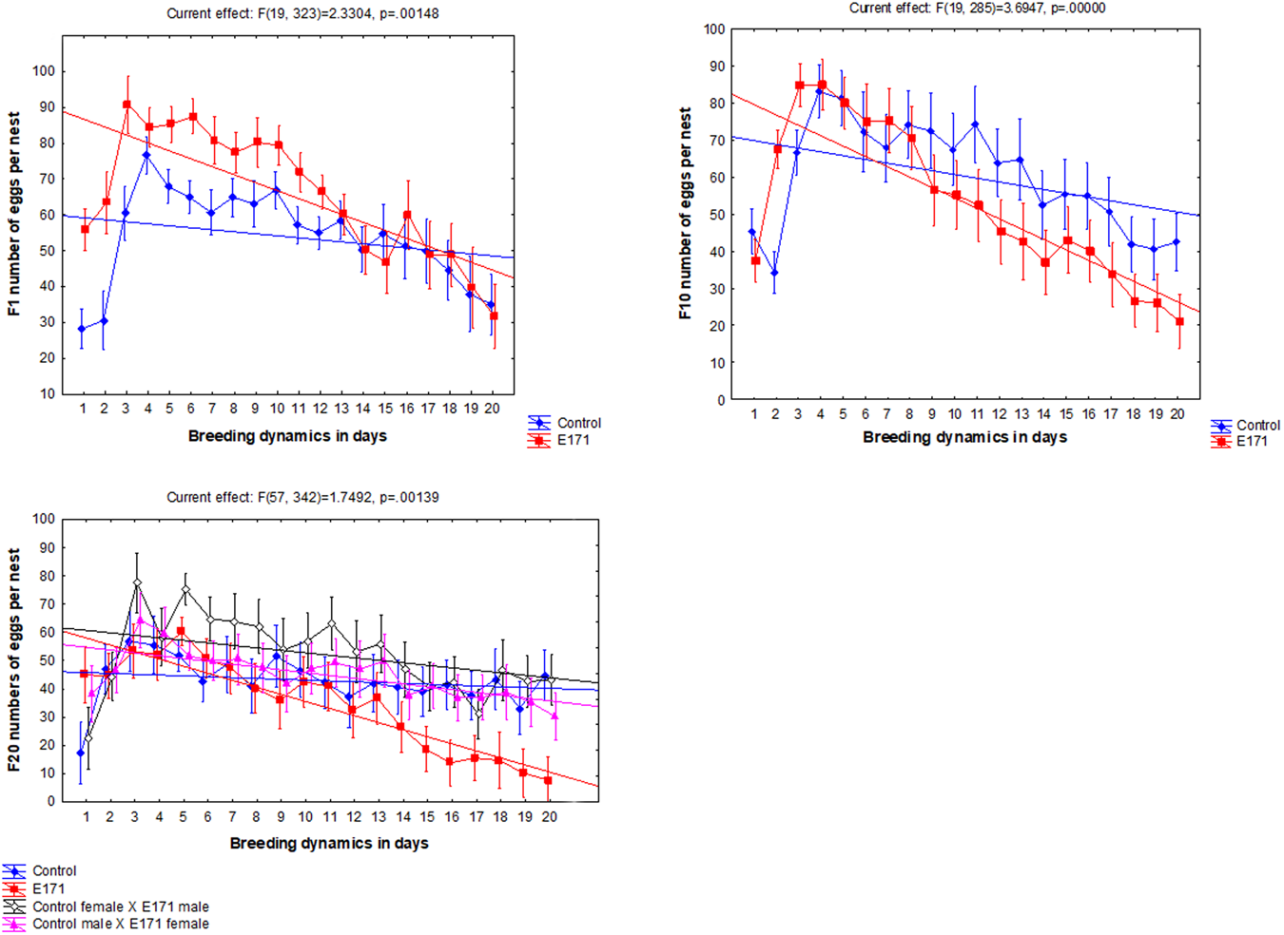
Least square means of *D*. *melanogaster* fecundity across generations and across breeding intervals within generations. On the X axis each number denotes a consecutive day on which the eggs were laid by the same parents. Parents were approximately 6 days post eclosion old when they began to breed. Whiskers represent the standard error of the mean.

On average, the fecundity of the first breeding instance of virgin females was also statistically higher in the E171 treatment (Mann-Whitney U-test: p < 0.05; N = 195 per group). Irrespective of their generation, the control virgin flies laid 32.6 ± 2.8 eggs per nest, while the E171 virgin flies laid 37.3 ± 3.0 (mean ± 95% CI). Fecundity was influenced by the treatment, generation number, and by treatment * generation interaction (RANOVA: F = 6.46, F = 7.96, and F = 4.58; p < 0.05, p ≪ 0.001, and p ≪ 0.001 for treatment, generation number, and treatment * generation interaction, respectively), but the pattern was not straightforward. Periods of higher and lower fecundity alternated between the control and treatment groups during the experiment (Fig. 1B). However, repetitive breeding of the same females and males over the course of 20 days resulted in significantly lower fecundity in the E171 treatment group over time. This effect was even more apparent in subsequent generations. Virgin E171 females had higher fecundity, usually during the first few matings, followed by a sharp decline in fecundity after 3–5 days of consecutive mating when compared to the control (RANOVA; time * treatment interaction, p < 0.001 for each generation) (Fig. 2). It is interesting to note that this effect was persistent only for the E171 treatment groups, not for crossings between E171 and control flies. In F_20_, where the effect for E171 group was the most obvious, the crossing of males and females from the E171 and control groups reverted the fecundity back to normal control values and the interaction of time * treatment was no longer present (RANOVA; df = 38, F = 1.1, p ≫ 0.05).

**Figure 3:**
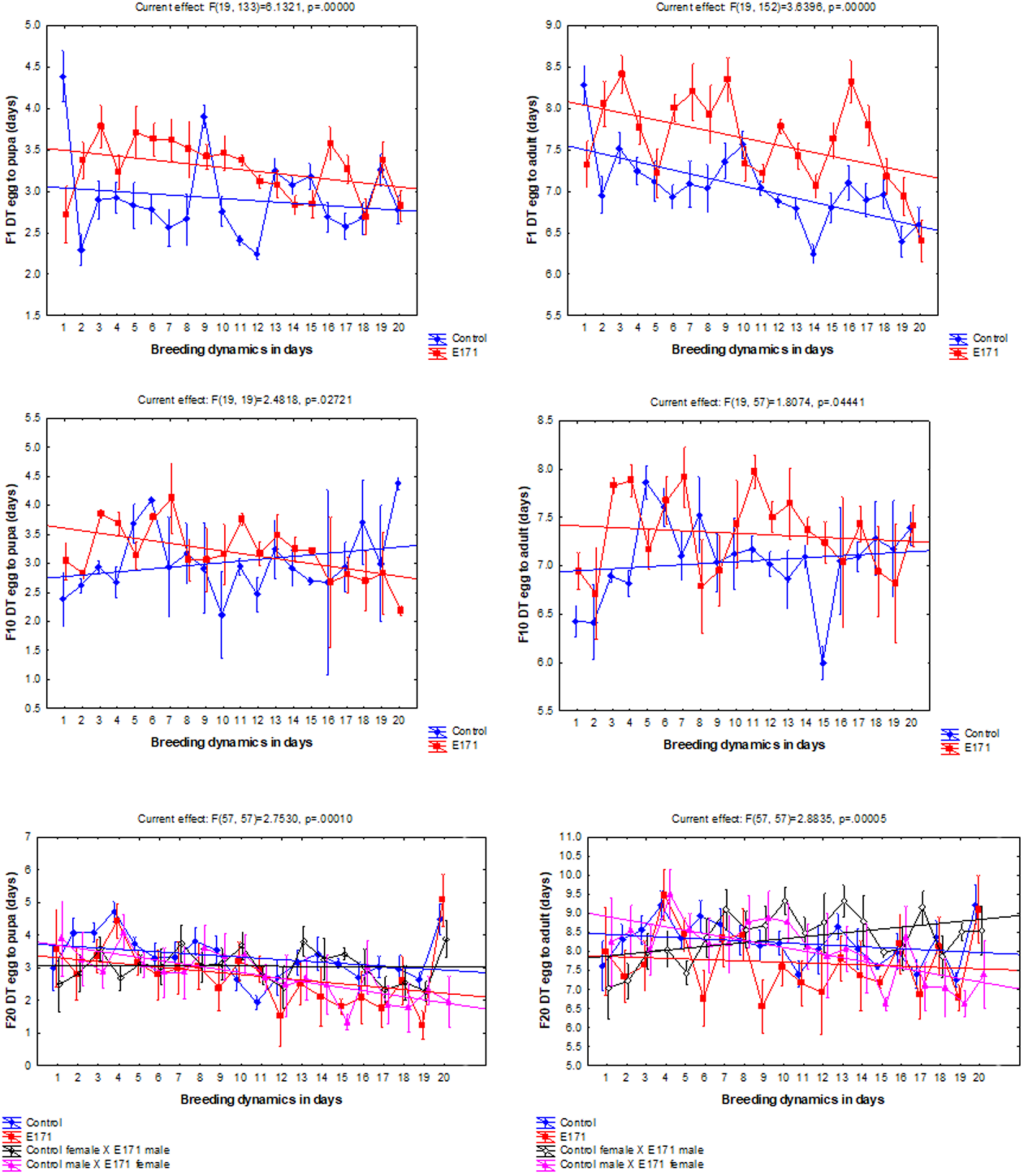
Least square means of *D*. *melanogaster* developmental time (DT) across generations and across breeding intervals within generations. On the X axis each number denotes a consecutive day on which the eggs were laid by the same parents. The parents were approximately 6 days post eclosion old when they began to breed. Whiskers represent the standard error of the mean.

The developmental time (DT) was affected by the treatment and it was dependent on the generation number. The effect over generation time can be best represented by a U-shaped toxicology curve. At the beginning, in the F_1_ generation, the addition of E171 to the diet significantly increased egg to pupa DT from 2.97 ± 0.74 to 3.14 ± 0.66 days (mean ± SD) (t-test, df = 354, p < 0.05), as well as egg to adult DT from 7.14 ± 0.72 to 7.49 ± 0.79 days (mean ± SD) (t-test, df = 354, p < 0.001). Later, however, in generation F_10_ there was no difference in DT (Table 1), while at the end in generation F_20_ the effect was reversed and the E171 fed flies had shorter DT than the control flies (ANOVA: df = 3, F = 4.88, p < 0.05). Interaction between the treatment and time when the eggs were laid (age of parents) was always a significant factor which influenced DT as outlined by RANOVA (Fig. 3). In general, according to the linear fit model, as the parents were growing older, the DT of their offspring was getting slightly shorter, although the relationship is not straightforward. As the effects of the treatment across the generations significantly changed from prolonging to shortening the DT, the age of the parents seems to contribute to the observed effect in a complex way.

**Table 1:** Developmental time (DT) of *D*. *melanogaster* in days (mean ± SD).

Generation	Control		E171		Control male × E171 female		Control female × E171 male	
	DT egg to pupa	DT egg to imago	DT egg to pupa	DT egg to imago	DT egg to pupa	DT egg to imago	DT egg to pupa	DT egg to imago
F1	2.97 ± 0.74	7.14 ± 0.72	**3.14 **±** 0.66**	**7.49 **±** 0.79**				
F10	2.91 ± 0.66	6.97 ± 0.63	2.86 ± 0.76	6.95 ± 0.70				
F20	3.23 ± 0.78	8.09 ± 0.75	**2.82 **±** 0.84**	**7.75 **±** 0.79**	**2.80 ± 0.64**	8.07 ± 0.68	**2.87 ± 0.80**	8.18 ± 0.91

Bold values indicate that the effect is statistically significant when compared to the corresponding control.

**Figure 4:**
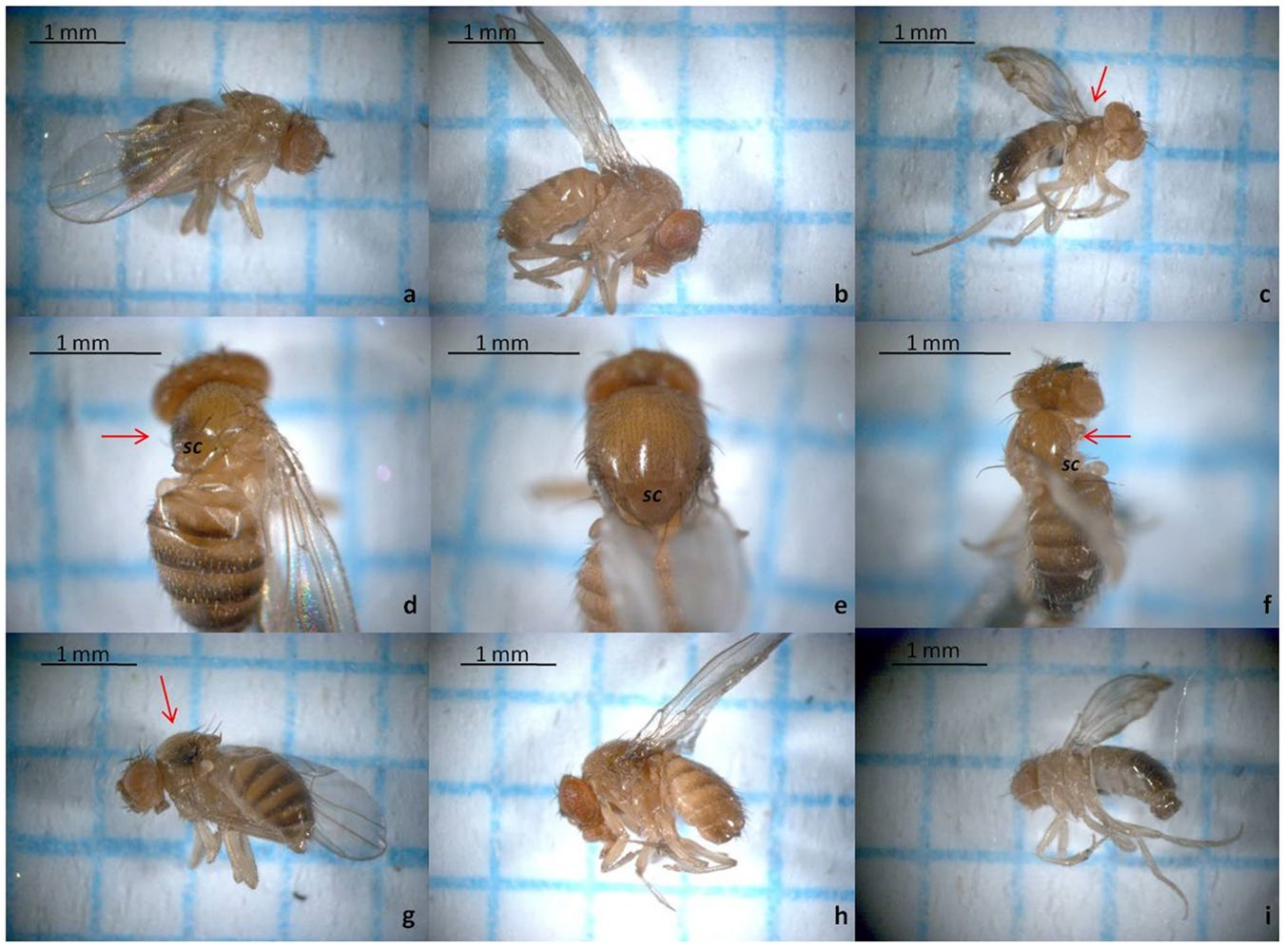
Phenotype of the aberrant flies. (**a**,**d**,**g**) Lateral and dorsal view of the aberrant female fly from E171 treatment. (**c**,**f**,**i**) Lateral and dorsal view of the aberrant male fly from E171 treatment. (**b**,**e**,**h**) Lateral and dorsal view of the normal fly. Red arrow shows wingless and distorted side of the thorax. ***sc*** denotes *scutellum* part of thorax. (**a**–**c**,**g**–**i**) Magnification 25×. (**d**–**f**) Magnification 40×. Bars show 1 mm.

Aberrant phenotypes appeared occasionally and exclusively in the E171 treatment, however, always at a low number (<0.1% of all the flies screened). These aberrant phenotypes are described in details in Fig. 4, and are the same as those discovered in our previous preliminary study^17^. The thorax of the aberrant individuals is curved toward the wingless side. The *prescutum* and *scutum* of the thorax are visibly reduced, while the *scutellum* is curved toward the wingless side of the body. The males have more severe deformities than the females (Fig. 4). An attempt was made to cross mate aberrant males and aberrant females with normal phenotype flies in order to isolate the aberrant strain on a control feeding medium. During the experiment, however, aberrant males and females were not available simultaneously and therefore could not be crossed. Aberrant males had visible difficulties mating with the normal virgin females and mating occurred only after the wings of the normal virgin females were clipped. The offspring of either aberrant male/female X normal phenotype did not contain any aberrant phenotypes. The F_1_ generation was then self-crossed and then F_2_, F_3_, F_4_, and F_5_ were also self-crossed. No aberrant phenotype was encountered again in the descendants of either a male or female aberrant parent. Therefore, it was concluded that this is not a hereditary feature caused by a mutation, but is rather an occasional metamorphosis or developmental error induced by titanium dioxide nanoparticles.

Both the larvae and adult flies ingested E171 from the medium, which was confirmed by means of ICP-MS. The accumulated concentration of TiO_2_ in the larvae was 3.84 ± 0.68 mg kg^−1^ bodyweight (mean ± SD), while the adult flies accumulated 0.45 ± 0.17 mg kg^−1^ bodyweight, which is significantly less (t-test, p ≪ 0.001). It is interesting to note that adult flies are likely to have a faster elimination rate of titanium. Based on the experiment setup, the larvae were supposed to eat 1.35 X more E171 than the adults, however this cannot account for an accumulated amount that is 9-10 X higher. Therefore, a difference in the elimination rate of E171 is the only plausible hypothesis. In support of this hypothesis are the results of the background concentration of elemental titanium from the environment in the control samples that was four times lower in the adults than the titanium concentration in the larvae.

The results obtained using the alkaline version of the comet assay are shown in Table 2. In F_1_, F_10_, and F_20_ the results indicate that E171 induced a significant increase in the total comet scores (18, 20.4 and 25.4, respectively) compared with the appropriate negative controls (10.5, 8.1 and 10.1, respectively). The genotoxic effect of E171 was statistically significantly higher in each subsequent generation compared to the previous one. The positive control carried out with the EMS showed a clear response, with a total comet score of 94.2. The data also showed that comet class 0 was the most frequent among larvae in the negative controls and larvae treated with E171. Comets in classes 3 and 4 were found only among larvae treated with EMS.

**Table 2:** Genotoxicity of E171 in *D*. *melanogaster* third instar larvae in F1, F10 and F20 generation.

	Comet class					Total comet score
	0	1	2	3	4	
EMS	40.4 ± 1.2	34.6 ± 0.8	17.3 ± 2.3	5.8 ± 0.6	1.9 ± 0.00	94.2 ± 1.3^bcdefg^
			F1 generation			
Negative control	89.5 ± 0.71	10.5 ± 1.7	0.00 ± 0.00	0.00 ± 0.00	0.00 ± 0.00	10.5 ± 1.7^ac^
E171	85.8 ± 5.1	10.4 ± 3.4	3.8 ± 2.5	0.00 ± 0.00	0.00 ± 0.00	18 ± 0.8^abcdfg^
			F10 generation			
Negative control	91.9 ± 1.1	8.1 ± 1.2	0.00 ± 0.00	0.00 ± 0.00	0.00 ± 0.00	8.1 ± 1.2^abd^
E171	86.8 ± 0.7	6 ± 3.1	7.2 ± 2.9	0.00 ± 0.00	0.00 ± 0.00	20.4 ± 1.5^abcdeg^
			F20 generation			
Negative control	92.8 ± 0.52	4.3 ± 0.31	2.9 ± 0.7	0.00 ± 0.00	0.00 ± 0.00	10.1 ± 0.54^ac^
E171	83.7 ± 0.8	7.2 ± 1.01	9.1 ± 0.5	0.00 ± 0.00	0.00 ± 0.00	25.4 ± 0.83^abcdef^

^a^*p* < 0.05 when compared with the positive control group.

^b^*p* < 0.05 when compared with the negative control group in the F1 generation.

^c^*p* < 0.05 when compared with the negative control group in the F10 generation.

^d^*p* < 0.05 when compared with the negative control group in the F20 generation.

^e^*p* < 0.05 when compared with the E171 in the F_1_ generation.

^f^*p* < 0.05 when compared with the E171 in the F_10_ generation.

^g^*p* < 0.05 when compared with the E171 in the F_20_ generation.

**Figure 5:**
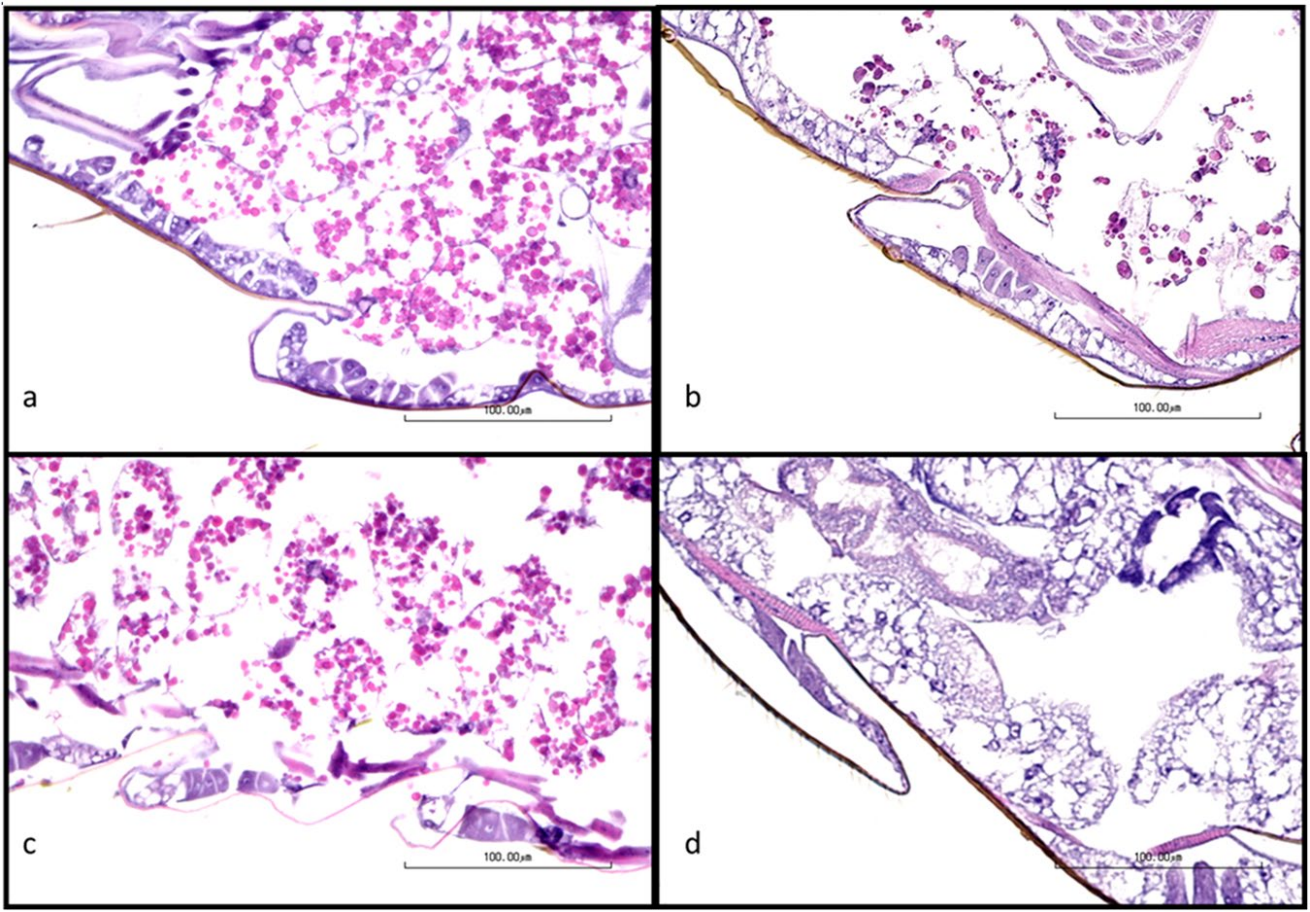
Photomicrographs of abdominal fat bodies of representative control (**a**,**c**) or E171 TiO_2_-fed (**b**,**d**) adult flies. Note fewer intracytoplasmic proteinaceous globules in fat body trophocytes in flies fed E171 TiO_2_ (groups **b**,**d**). Hematoxylin and eosin staining.

**Figure 6:**
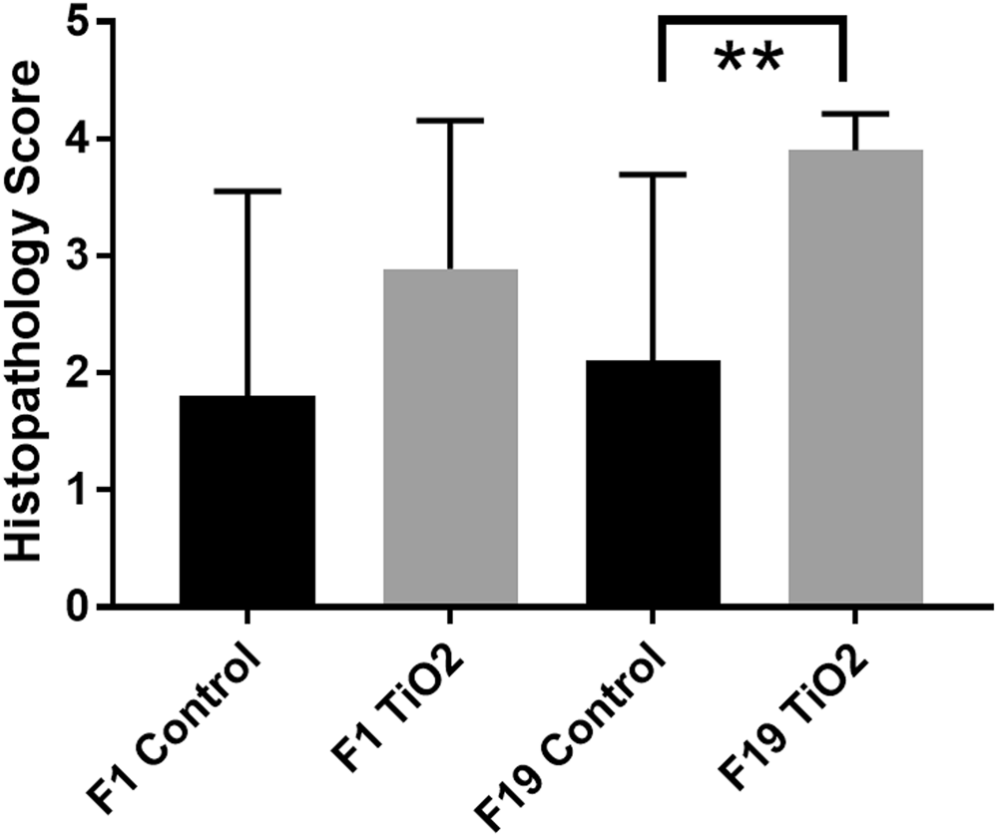
Graphical representation of semi-quantitative histopathology scores for loss of protein globules in fat body cells of control or TiO_2_-fed adult flies from F1 or F19 generations. **Indicate statistically significant difference, p = 0.0262, Tukey’s multiple comparison test.

Among adult flies, there was variation in the amount of intracytoplasmic protein granules in trophocytes (adipocytes) of the fat body (Figs 5 and 6), with more loss of intracytoplasmic protein granules in flies fed E171 TiO_2_. Statistical comparison of histopathology scores for loss of protein granules in fat body trophocytes were different among groups (p = 0.0067, ANOVA). In post-testing, a statistical difference in fat body morphology was present between F_19_ generation control and E171-fed flies (p = 0.0262, Tukey’s multiple comparisons), as well as between F_1_ control and F_19_ E171-fed flies (p = 0.0074, Tukey’s multiple comparisons). Other histologic differences between groups were not observed in adult flies. Differences in histologic features were not observed between groups of larvae.

## Discussion

In the present study, F_1_ flies exposed to 0.014 mg mL^−1^ of E171 showed the same pattern of delayed DT as the flies exposed to 0.02 mg mL^−1^ of E171 in our previous preliminary study^17^. The marked delay in the development of fruit flies exposed to TiO_2_ nanoparticles is concentration dependent^17,19^. The delayed DT of fruit flies exposed to E171 is due to the oxidative stress caused by TiO_2_, the change of expression in the genes involved in response to oxidative stress, and energy balance partitioning^17^. In later generations, DT was no longer delayed but was in fact shortened and in the end resulted in significantly shorter F_20_ DT of E171 fed flies compared to the control (Table 1; Fig. 3). This effect can be seen as one of the classical adaptations of the fruit fly population to environmental contamination with metals^20^. In fruit flies, shorter DT is an advantage when stress is encountered, since the larval stage is exposed for shorter amounts of time to the stressful environment. The initial higher egg to adult viability and fecundity of fruit flies reared on E171 medium is likely an example of directional selection in a polluted environment. The more energy invested earlier in reproduction, the more likely it is that the parent will produce offspring before succumbing to the pollutant. Higher resistance of individuals from contaminated sites, by means of higher fecundity, as opposed to controls, has been documented numerous times, both in fruit flies and other organisms^20–23^, especially when the concentration of the pollutant is low. This, however, does not mean that all or the majority of populations exposed to pollutants will develop higher resistance, or exhibit higher fecundity. In fact, reduced fecundity is a commonly observed negative effect after exposure to a pollutant. If the breeding dynamics are observed, only E171 fed females from F_1_ managed to keep significantly higher fecundity throughout adulthood compared to the controls. In both F_10_ and F_20_ E171 fed females there is a sharp decline in fecundity after 3–5 days of consecutive mating when compared to the control (Fig. 2). This is likely the result of a long-term adaptive effect against E171 toxicity. If adaptation to E171 toxicity is geared toward shorter DT and faster growth through the generations, then loss of fecundity in later generations is in agreement with the trade-off that more available energy would be allocated toward fast development/growth and less toward late reproduction^24^.

Both the larvae and adults ate E171 from the medium as confirmed by ICP-MS analysis. In theory, both larvae and adults were supposed to have the same concentration of TiO_2_ in their bodies since they were fed with the same amount of TiO_2_. However, the larvae contained approximately 10 X more TiO_2_ compared to the adults. Even in the control samples, the larvae contained 4 X more elemental titanium from the environment than the adults. This means that the adults have a much more efficient way to eliminate titanium and TiO_2_ from the body. Previously, we reported that if only the fruit fly larvae are fed with E171 then the emerging adults will be free of any TiO_2_ burden accumulated by the larvae^17^. This is mainly because the larvae of flies defecate before pupation as well as immediately after eclosion. As a result, in dietary studies, the concentrations of administered metals are often significantly smaller in various species of adult flies compared to larvae^25,26^. However, if the same adults of the fruit flies are fed with TiO_2_ in continuity after eclosion, as was the case in the present study, then the adults should have a similar concentration of TiO_2_ in their bodies to the larvae. Since this is not the case, adult fruit flies in fact eliminate TiO_2_ from their bodies at a much faster rate than the larvae. Therefore, the larvae bear a significantly higher risk associated with E171 exposure than the adults do. Alternatively, *Drosophila* larvae particularly show a great voracity behavior between 1st and 3st instar and therefore a sudden increase in ingesting of food media is expected, suggesting that the higher content of TiO_2_ in the bodies of larvae may be attributed simply to this behavior. This voracious behavior could somewhat explain 4 X higher Ti levels in control larvae as compared to adults, yet it can not be fully attributed to the 10 X more TiO_2_ compared to the adults in the experimental group.

TiO_2_ is known to cause oxidative stress in cells, which is a basis for genotoxicity^27^. The genotoxicity of TiO_2_ has been demonstrated by means of the Ames test, comet assay, micronucleus assay, sister chromatic exchange assay, mammalian cell gene mutation assay, and the wing somatic mutation and recombination assay, and it was also systematically reviewed recently^27^. Both positive and negative results were reported, with a greater number of positive responses in the *in vitro* systems than *in vivo* and in the assays measuring DNA and chromosome damage than the assays for gene mutation^27^. Exposure of fruit flies to TiO_2_ also resulted in genotoxicity^28^, although in some cases there was no increase in genetic damage^29^. Specifically, TiO_2_ was not able to induce genotoxic activity in the wing spot test of *Drosophila*^28,29^, while the alkaline comet assay revealed DNA damage after TiO_2_ exposure^28^. The latter is in agreement with the results of the current study. Different results on genotoxic evaluation of TiO_2_ nanoparticles in *Drosophila* can be related with genotoxic system assay employed. For example, SMART assays can detect fixed genetic damage (i.e. mutations) in wing somatic cells of fruit flies, while comet assay is a tool that detect primary DNA damage that potentially could be convert into mutation if it cannot be repaired by cells. Therefore, dissimilar results can be related simply with differences in genotoxic end-points of each system assay used. The genotoxicity of the E171 grade has been tested only recently, and according to the results, E171 has even higher potential for a generation of reactive oxygen species and it induced higher cytotoxicity/genotoxicity than nanoparticulated and microparticulated fractions of regular TiO_2_ alone^30^. To our knowledge, the present study is the first study to investigate the genotoxicity caused by E171 (or any other TiO_2_) in a multigenerational study. The results showed that the genetic damage caused by E171 increases with each increasing generation exposed to E171 and it is significantly higher when compared to the control. The comet assay was performed on somatic cells and not on germinative cells. Therefore, it is unknown whether the observed increase in genetic damage across the generations is due to the accumulation and transfer of damaged DNA to subsequent generations. Alternatively, subsequent generations may have suffered from a decrease in the defense mechanism ability against oxidative damage due to tradeoffs with other physiological processes. Nano-TiO_2_ has a strong binding affinity to DNA^31–34^; it can structurally alter DNA after binding^31^ and can act as a clastogen^33^. Interestingly, although nano-TiO_2_ induced ROS does cause genotoxicity, the primary cause of genotoxicity is not ROS but the direct binding activity of nano-TiO_2_ to DNA^32^. Genotoxicity was likely responsible for the development of the aberrant phenotypes. These phenotypes were the same as those previously observed in our preliminary study^17^. They were also somewhat similar to the isolated *Drosophila* mutants after exposure to gold nanoparticles^35^ because in both cases the thorax of the aberrant individuals had similar curvature, while the *prescutum* and *scutum* of the thorax were visibly reduced. Exposure to various types of nanoparticles (Ag, TiO_2_, Co, Au, ZnO, zirconia, and hydroxyapatite nanoparticles) is known to cause aberrant phenotypes with aberration observed in the wings, bristles, thorax, abdomen, eyes, cuticle development, and pigmentation^36^. While some of these aberrations are caused by mutations and are passed down onto successive generations^35,37^ not all of them are inheritable. In a *Drosophila* model, nanoparticles can also cause somatic mutations^38,39^, induce alteration in a signaling pathway^40^ resulting in a defective adult fly^36^, or cause developmental abnormalities^41,42^, any of which may be responsible for the aberrations observed. However, in the present research the aberrant phenotypes were likely caused by developmental defects induced by E171, since phenotypic features were not transferred to the progeny even after 5 generations of consecutive crossbreeding. Missing wings and thoracic deformations may have been caused by erroneous stages of development of the wing imaginal disc. TiO_2_ nanoparticles are known to cause imaginal disc damage^28^. Previously, it was suggested that the abnormal wing phenotypes observed in fruit flies exposed to TiO_2_ were caused by wing imaginal disc alteration during the developmental stage^19^. Another phenotypic aberration is missing bristles^19^. In the present research, bristles were not analyzed. Wing formation involves various signaling pathways that guide development of the imaginal disc. One common pathway is Notch signaling^43^. While nanoparticles are used in medical research to target and suppress Notch signaling^44^, we are not aware of any study proving that TiO_2_ can suppress Notch, although it has been suggested recently as a possibility^36^.

The insect fat body functions in nutrient storage and metabolism regulation, similar to adipose tissue and liver of vertebrates. Fat body cells, the trophocytes, contain intracytoplasmic lipid droplets that contain lipids and proteins, the type and amount varying with metabolic state^45–49^. After 19 generations ingesting a diet containing TiO_2_, adult flies had reduced amounts of protein globules in fat body cell cytoplasm. This may reflect decreased production or uptake of proteins or by increased loss of proteins, possibly through autophagy, dissociation from lipid droplets, secretion, or other process. The metabolic effects of this morphologic change are not known, but proteins critical for the development of the imaginal disc and wing structure are expressed and active in the fat body^50–54^ and may contribute to the rare aberrant phenotypes we observed.

## Conclusion

In conclusion, dietary exposure of 20 consecutive generations of *D*. *melanogaster* to an estimated daily human consumption dose of E171 resulted in significant alterations in normal developmental and reproductive dynamics, reduced fecundity after repetitive breeding, increased genotoxicity, appearance of aberrant phenotypes and morphology. These effects are considered to be among the classical adaptations of the fruit fly population to a stressor. Shorter developmental time coupled with higher fecundity and egg to adult viability in virgin females but reduced fecundity at any subsequent mating events is a pattern that was gradually observed over the 20 generations of flies and it exhibits marks of adaptive evolution and directional selection. This is in agreement with the trade-off hypothesis that more of the available energy would be allocated toward faster development and early reproduction and less toward later repetitive reproduction events. Shorter developmental time is an advantage when stress is encountered since the larval stage is exposed for a shorter amount of time to the stressful environment. In addition, larval stages had a higher risk of sustaining damage from E171 as they had a slower elimination rate and accumulated 10 X more TiO_2_ compared with adults. This is particularly worrisome, as among the human population children tend to consume higher daily concentrations of E171 than adults do. Aberrant phenotypes were likely caused by developmental defects induced by E171, since the phenotypic features were not transferred to progeny even after 5 generations of consecutive crossbreeding. Therefore, exposure to E171 during early development carries a higher risk of toxicity, and again in the human population fetuses and young children would be the most endangered cohort. The fact that the daily human consumption concentration of E171 is able to interfere with and influence the physiological, ontogenetic, genotoxic, and adaptive processes of fruit fly certainly raises a safety concern.

## Material and Methods

### E171 TiO_2_

In the present research, the same stock suspension of E171 TiO_2_ from our previous experiment^17^ was used. Approximately 30% of the particles in suspension are <100 nm in diameter. Before its use, the stock suspension was tested for bacterial and fungal contamination by means of inoculum on standard bacterial/fungal media. The stock suspension was confirmed to be sterile as no microorganism grew in the Petri dishes. The E171 (C.I. 77891) was manufactured by a commercial supplier - Fiorio Colori of Italy. The same stock suspension was used in order to provide a more reliable comparison between the previous and present experiments. Previously, we provided a comprehensive physico-chemical characterization of this particular batch of E171^55^. In summary, E171 is at least 99% pure, and has an anatase crystalline structure. The specific surface area of E171 is 6.137 m^2^ g^−1^, the pore volume is 0.123 cc g^−1^, and the pore diameter is 2.97 nm. It has a Ti_2_p3/2 peak at 463.8 eV and 2p1/2 peak at 458.0 eV, while a small shoulder at 532.5 eV implies that the surface is partially covered with hydroxide groups. The particles are amorphous-spherical-like with sharp and well-defined clean edges. The mean particle size ± standard error of the mean (SEM) is 167 ± 50 nm.

### D. melanogaster care and housing

The fruit flies, Oregon-R-C (Stock number 5) wild-type strain (Bloomington Drosophila Stock center at Indiana University, USA) were housed *en masse* at an optimum density as recommended by Bloomington Drosophila Stock Center, Indiana, USA. They were maintained at optimal conditions of 25 ± 1 °C, a 12/12-hour day/night regime and 60% humidity. A standard cornmeal-based feeding medium was used, consisting of 10% cornmeal, 9% sugar, 2% yeast and 2% agar with the addition of the fungicide Nipagin (2.50 mg mL^−1^) (Alfa Aesar GmbH & Co KG, Germany) diluted in 96% EtOH.

### Exposure concentration

The diet of each experimental group contained 0.014 mg mL^−1^ of E171 in the feeding medium, in addition to the above-mentioned ingredients, and the E171 was homogenously mixed into it. Flies were not on a nutrient restriction diet and therefore addition of E171 could not interfere with the calories intake. Briefly, before adding the E171 suspension to the liquid feeding medium, the stock suspension was sonicated for 2 min in a standard Fisherbrand sonicator bath to disperse the particles. Then, the E171 stock suspension was added to the warm, freshly prepared liquid feeding medium and stirred to homogenize. The liquid feeding medium was poured into 50 mL vials to cool down, and yeast extract was added to the surface. A concentration of 0.014 mg mL^−1^ E171 in the feeding medium was chosen in order to provide an average exposure of 20 mg kg^−1^ larvae/adult fly bw per day. An exposure concentration of 20 mg kg^−1^ larvae/adult fly bw per day was chosen, as it is believed to be close to the maximum possible exposure scenario in humans. For the maximum level exposure assessment scenario, the mean exposure estimates ranged from 0.4 mg/kg bw per day for infants and the elderly to 10.4 mg/kg bw per day for children, while at the 95th percentile, exposure estimates ranged from 1.2 mg/kg bw per day for the elderly to 32.4 mg/kg bw per day for children^7^. An adult *D*. *melanogaster* weighs on average 0.8 mg^56^ and eats 1.5 µL of feeding medium per day^57,58^, which is 1.7 times its body mass. A larva has an average mass of 1.8 mg^59^, and can eat about 3 µL of feeding medium per day assuming it also eats 1.7 X its body mass per day. This means that in order for an adult fly to be exposed to an E171 concentration of 20 mg kg^−1^ bw per day the amount of E171 in the feeding medium should be 0.016 mg mL^−1^. For the same level of larvae exposure, it should be 0.012 mg mL^−1^. In practice, it is not very plausible to prepare and maintain large numbers of different feeding mediums for 20 generation of flies and since the two concentrations differ by only 30%, all feeding media were prepared with an average concentration of 0.014 mg mL^−1^. Therefore, the adults were effectively exposed to 17 mg kg^−1^ bw per day of E171 and the larvae to 23 mg kg^−1^ bw per day of E171. This is also in line with the current understanding that children are exposed to higher levels of E171 than adults^7^.

### Experimental setup

The experiment included one parental generation (F_0_) and 20 filial generations (F_1–20_) of *D*. *melanogaster*. The F_0_ was chosen randomly from the *en masse* population and consisted of young adults, approximately 6 days old. These adults were randomly divided into 2 groups. Each group consisted of approximately 50 males and 50 females, which were housed separately. One group served as a negative control, while the second group received E171 in the feeding medium. From this point onward, these two lines were never crossed until F_20_, and adults from the E171 or control groups mated separately. The F_0_ cohort was allowed to feed for 3 days, after which 10 males/females from each group were paired randomly. Each pair was kept separately in a 50 mL tube. The F_1_ generation was formed from the first batch of eggs from each pair. The F_0_ adults were removed from the tubes within 8 h of laying their eggs, while the eggs remained in the tubes and were incubated until the larvae hatched. Within the first 24 h of incubation time, all of the eggs were counted before hatching. Incubation was continued until the larvae reached the pupa stadium, then all of the pupae were counted. Immediately after eclosion, the adults were counted, separated by gender and transferred to new 50 mL tubes with E171 or control feeding medium in order to prevent uncontrolled mating. Once the adults reached approximately 6-days–post eclosion, 10 random male/female pairs per control or treatment were paired and each pair was transferred to a new 50 mL tube with fresh medium. The paired males and females always originated from a different parent in order to avoid inbreeding and a bottleneck effect. The pairs mated for 20 days (actual post eclosion age in days 6–25), and each day adults were transferred to a new medium, while the eggs were counted and observed every day until eclosion. The developmental time - DT (larva to pupa; larva to adult) was calculated for each of the 10 replicas for each day according to the following formula

$$DT=\sum _{d=1}^{x}\,{n}_{d}\,\ast \,d/{n}_{t}$$

where n_d_ is the number of pupating larvae/emerging flies d days after the eggs were laid, and n_t_ is the total number of individuals pupating/emerging at the end of single generation experiment. In some cases, a female would lay unfertilized eggs or it would lay too few eggs. Additionally, some females would stop laying eggs completely after a certain day. If a batch of eggs produced <10 larvae/adults at the end of the cycle it would be excluded from calculation of the DT, as the low number of pupating/emerging individuals would potentially skew the results. The F_2_ cohort was formed from the eggs of the F_1_ generation that were laid on the 1st day (actual age of parents was 6-days-post-eclosion), as *D*. *melanogaster* is then at its full reproductive maturity^60^. The same procedure was repeated until F_20_. A pair of flies would occasionally not produce eggs on the 1st day, and in that case the first eggs that were produced on the next days were counted and used for creation of the next F generation.

Due to the high number of mating pairs, and the number of eggs, transfers to new mediums, etc., the daily DT was calculated only for generations F_1_, F_10_, and F_20_. Fecundity and egg to adult viability was calculated for each generation. In addition, these were calculated for each of 20 consecutive days of eggs laid in generations F_1_/F_10_/F_20_. Sometimes, in generations F_1_/F_10_/F_20_, the male or female would die before the 20 days of laying eggs had finished. If that was the case, the missing mate was not replaced and data were recorded only until the last day that both the male and female were alive.

The F_20_ generation had a slightly different experimental design. In addition to the standard setup used in the F_1_ and F_10_ generations, F_20_ contained two extra treatment groups that consisted of control males crossed with E171 females; and E171 males crossed with control females. The aim of the crossing was to determine if a long multigenerational exposure to E171 resulted in a gender-specific reproductive/developmental effects. Due to the crossings, and therefore the larger number of treatment groups, the number of replicas for F_20_ had to be reduced to 5–7 per group.

### Genotoxicity

The genotoxicity of E171 TiO_2_ was evaluated *in vivo* in the anterior midgut of *D*. *melanogaster* using the alkaline version of the comet assay. Phosphate-buffered saline (PBS) without calcium and magnesium, agarose for DNA electrophoresis, low-melting point agarose (LMA), and collagenase were obtained from Alfatrade Enterprise D.O.O.; methyl 4-hydroxybenzoate and ethyl methanesulphonate (EMS) were purchased from Sigma-Aldrich, St. Louis, MO, USA. In order to determine the potential for E171 to damage DNA in somatic cells across generations, the larvae of *D*. *melanogaster* were fed with the medium containing 0.014 mg mL^−1^ of E171, as previously described. EMS (1 mM in PBS) was used as a positive control. Genotoxicity was evaluated in generations F_1_, F_10_, and F_20_. The comet assay was performed as previously described^61^, with minor modifications^62^. Immediately before use, slides were stained with 80 µL of ethidium bromide (20 µg mL^−1^). The images were visualized and captured with the 40x objective lens of a Nikon fluorescence microscope (Ti-Eclipse) attached to a CCD camera. One hundred randomly selected cells (50 cells per two replica slides) were analyzed per treatment. The comets were analyzed by means of a visual scoring method^63^ and the total comet score was calculated according to^64^. The results were expressed as mean ± SEM and a statistical evaluation of the data was carried out by means of one-way analysis (ANOVA) using the SPSS statistical software package, version 13.0 for Windows. The significance level was set at p < 0.05.

### Confirmation of E171 ingestion

The flies were housed and fed as described above. Half of the larvae per treatment or control group were collected for inductively coupled plasma mass spectrometry (ICP-MS) shortly before turning into pupae, while the other half were allowed to develop into adults. Emerging adults were fed for an additional 6 days before collection for ICP-MS. One gram of larvae and 1 g of adults were collected from the control or E171 groups per replica. A total of 5 replicas were included. The samples were weighed and preserved in 70% ethanol until further ICP-MS analysis. The samples were digested and processed in the same way as in our previous study^65^. Samples from the present study were processed in parallel with samples from our previous study^65^, and therefore shared the same recovery for titanium of 94.7 ± 1.1% (N = 3) from standard reference material (mussel tissue, SRM 2976, NIST) as we reported previously. Furthermore, standard reference material (oyster tissue, SRM 1566b, NIST, Gaithersburg, Maryland, ZDA) containing 12.24 + 0.39 mg/kg of Ti was digested and analyzed in the same way as the samples. The Ti concentration in the digested SRM 1566b was determined to be 12.25 + 0.44 mg/kg (N = 3). The E171 intake by the larvae or adults was calculated using the following equation: E171 conc. = (Ti conc. of the experimental sample − background Ti conc. of the control) × mass ratio of E171/Ti. The mass ratio of E171/Ti = 1.6684.

### Histopathology

Adult flies and larvae from the F_1_ and F_19_ generations were euthanized in ethanol, fixed in 10% neutral buffered formalin, processed routinely into paraffin blocks, cut in step sections at 5 microns, stained with hematoxylin and eosin, and examined microscopically under bright-field conditions. At least 10 sections from 10 adults and 10 larvae were examined microscopically. Multiple organ systems of the adult were evaluated, including the exoskeleton, eye, central nervous system, mouthparts, salivary gland, foregut, midgut, hindgut, skeletal system, reproductive system, and fat bodies. Organ systems of the larvae evaluated include the exoskeleton, central nervous system, mouthparts, salivary gland, intestine, skeletal muscle and fat bodies. Tissue and cytomorphologic changes were recorded using a semi-quantitative severity scale from 0 to 5 where 0 = no lesion and 5 = severe lesion. Changes in the morphology of the abdominal fat body of adult flies was scored using the following criteria: 0 = >40% of fat body trophocyte volume is protein globules, 1 = 20–40% of fat body trophocyte volume is protein globules, 2 = 5–20% of fat body trophocyte volume is protein globules, 3 = rare intracytoplasmic protein globules, and 4 = no protein globules in fat body trophocyte cytoplasm.

### Statistics

Unless otherwise noted, Repeated factorial Analysis of Variance (RANOVA) was used to check the effects of the treatment, generation number, and treatment * generation number interaction effects using Statistica 12.0 software. Before analyses, the data were analyzed using the Kolmogorov Smirnov and Lilefors tests for normality. If assumption of normality was not met, data were Box-Cox transformed before running ANOVA. Student’s t-test was used to compare the ICP-MS data. The comet assay was analyzed with ANOVA using the SPSS statistical software package, version 13.0 for Windows. Histopathology scores were analyzed by ANOVA and Tukey’s multiple comparison tests using GraphPad Prism 7.04. Only a p value of <0.05 was considered statistically significant.

## Data Availability

The datasets used and/or analysed during the current study are available from the corresponding author on reasonable request.
